# Impact of landscape installation design on emotional loneliness: interaction style as a moderating factor

**DOI:** 10.3389/fpsyg.2025.1678736

**Published:** 2025-12-12

**Authors:** Mou Gong

**Affiliations:** Wuhan Business University, Wuhan, China

**Keywords:** landscape installation, user interaction, design elements, emotional loneliness relief, cultural, color, function

## Abstract

The social isolation measures implemented during the COVID-19 pandemic have exacerbated emotional loneliness, presenting significant challenges in the fields of public health and social psychology. This study examines the potential of landscape design in mitigating emotional loneliness. While prior research indicates that art therapy can reduce environmental loneliness and provides theoretical support for emotion-focused environmental interventions, systematic investigations into the role of landscape installations in alleviating emotional loneliness remain limited. Further exploration of their mechanisms and practical applications is necessary. Through case studies and questionnaire survey-based methods, this research evaluates how design elements—such as cultural references, color application, and function attributes—affect emotional loneliness relief. The findings demonstrate that design elements significantly enhance user interaction (*β* = 0.339, *p* < 0.01) and also directly alleviate emotional loneliness (*β* = 0.258, *p* < 0.01). User interaction, in turn, exerts a significant direct effect on emotional loneliness relief (*β* = 0.312, *p* < 0.01). Mediation analysis confirms that user interaction serves as a partial mediator, with the indirect effect supported by a 95% confidence interval excluding zero (95% CI: 0.065–0.148). Moreover, moderated mediation analysis indicates that interaction style moderates the path from user interaction to emotional loneliness relief. Specifically, the mediation effect is significant at medium and high levels of interaction style (Effect = 0.100–0.161). This study provides theoretical insights into the development of landscape design in relation to emotional health and offers empirical support for the implementation of landscape installations with psychologically supportive functions.

## 1 Introduction

### 1.1 Background and purpose

During the COVID-19 pandemic, stay-at-home measures and the widespread use of the internet led to an increased reliance on social media for communication. However, Kim (2015) found that this shift also led to increased anxiety among some individuals regarding face-to-face interactions. According to Cudjoe and Kotwal (2020), feelings of loneliness affect not only the elderly but also individuals across all age groups, particularly those who live alone or have mobility limitations. Findings by del Carmen García-Mendoza et al. (2024) suggest that psychological distress among young people increased significantly during the pandemic. Skoko et al. (2024) contend that loneliness, as a painful emotional state, results from perceived social isolation or unmet social needs. Additionally, Yan et al. (2024) observe that individuals with higher levels of loneliness during the early stages of the pandemic lockdown experienced more severe rates of depression.

Urban landscape spaces, as an extension of everyday life, play a crucial role in fostering social and artistic interaction. Masi et al. (2011) point out that outdoor activities and exposure to nature positively impact the quality of life for the elderly. In addition, McConatha et al. (2023) argue that natural spaces and greenery have significant positive effects on mental and physical health, contributing to mood enhancement. Caldarola (2020) suggests that since the 1960s, art has increasingly been applied to alleviate feelings of loneliness, particularly through landscape installations. These installations, by encouraging public participation, have become essential in the creation of artistic works. In landscape spaces, art installations serve as bridges between people and their surroundings, promoting community interaction and development through participant engagement, shared experiences, and thoughtful design.

Currently, research on landscape installations primarily centers on user interaction and environmental sustainability. Li and Ding (2014) proposed the concept of landscape interactive design, highlighting the symbiotic relationship between people and landscapes. With technological advances and the diversification of needs, the public’s expectations of urban design have steadily risen, especially in terms of seeking more meaningful interactive experiences. Permatasari et al. (2025) emphasizes the important role of ecological public art in sustainable urban development. Ji (2010) also underscores the critical role of renewable energy, resource efficiency, and green infrastructure in sustainable landscape design. Although Richmond-Cullen (2018) explored the role of art in alleviating loneliness, Aydın and Kutlu (2021) found that group art therapy, especially clay-based activities, can alleviate emotional loneliness in the elderly. Furthermore, Köse et al. (2024) argue that art therapy positively impacts the mental health of elderly nursing home residents. However, systematic research on the effects of landscape installations on emotional loneliness is still lacking.

Therefore, this study aims to explore the impact of design elements in landscape installations on emotional loneliness relief, with the objective of better understanding whether and how these design elements alleviate emotional loneliness. This research aims to provide a theoretical foundation for the future design of landscape installations that better meet people’s emotional and social needs.

To achieve this, the research objectives are as follows: first, to identify the key design elements in landscape installations that significantly contribute to emotional loneliness relief; second, to analyze how these design elements affect individuals’ emotional loneliness; and third, to propose practical design guidelines for future landscape installations that better address people’s emotional and social needs.

### 1.2 Definition of terms

#### 1.2.1 Design elements

Design elements, as perceptible attributes, systematically influence user emotions and behaviors, thereby shaping the overall user experience. Based on a thorough literature review, this study establishes four measurement dimensions for design elements: color, culture, function, and innovation. These dimensions are explored to understand their mechanisms in alleviating emotional loneliness within landscape installations.

First, according to Briggs (2023), color, as a key design element, encompasses three fundamental attributes: hue, brightness, and saturation. Zhou and Yang (2010) suggest that color serves both an expressive function in daily life and design practices and conveys specific emotional meanings, which influence user behaviors and attitudes.

The second dimension, culture, refers to the cultural symbols and narratives embedded in the design. Inam (2024) notes that cultural elements reflect the characteristics of specific ethnicities or regions, enhancing users’ cultural identity and sense of belonging, and deepening their emotional connection with the environment.

The innovation dimension focuses on creating novel experiences by challenging conventional design boundaries. Zhang (2021) argues that innovative designs often cross traditional limits in unique ways, stimulating user interest and engagement, which in turn enhances the appeal and novelty of the interactive experience.

Finally, functionality refers to the practical value of the design, directly addressing user needs and comfort. As Norman (2013) points out, functional design is a core component of user experience. It not only provides practical value but also significantly enhances the usability and practicality of the design.

#### 1.2.2 User interaction

Dong (2016) defines “user interaction” as a two-way process between individuals and their environment. In this process, users engage with the landscape through physical perception, forming emotional experiences that establish a bridge between design and emotion.

Based on a review of the literature, this study categorizes the evaluation metrics for user interaction into three main aspects: engagement, interaction fluidity, and emotional feedback. Engagement reflects the level of time, attention, and emotional investment users dedicate during the interaction. According to O'Brien and Toms (2008), high engagement indicates the effectiveness of the design in attracting and maintaining user interest. Interaction fluidity refers to the smoothness and naturalness of the interaction between users and the system. Norman (2013) emphasizes that good interaction fluidity enables users to complete tasks seamlessly, with the entire process being intuitive and efficient. Emotional feedback is used to assess the emotional responses generated by users during interaction. Preece et al. (1994) notes that this metric effectively reflects whether the design successfully triggers positive emotions and fosters an emotional connection with the user.

#### 1.2.3 Interaction styles

According to Hassanzadeh (2023), interaction style can be defined as the manner in which individuals engage with their environment. Based on the existing literature, this study categorizes the evaluation metrics for interaction style into four key aspects: auditory interaction, light and shadow interaction, dynamic interaction, and tactile interaction.

Kenwright (2020) points out that sound, in an interactive environment, not only enhances the perceptual quality but also plays a crucial role in guiding users’ emotions and attention. Similarly, McKeen et al. (1994) emphasize that auditory interaction helps to improve users’ spatial awareness and sense of involvement.

On the visual level, Burke (2002) suggests that light and shadow interaction is one of the key factors in enhancing audience engagement. Beyond the audiovisual dimensions, dynamic interaction, especially in scenarios like remote performances, provides users with new possibilities for interaction. For example, Kievit (1965) described a multimedia exhibition system where the audience could remotely adjust the music and lighting in the performance space, while performers could enhance their involvement in the exhibition space by controlling robotic arms.

In addition, tactile interaction, as a form of interaction based on the sense of touch, should not be overlooked. Altinsoy and Merchel (2018) note that such interaction typically relies on physical sensory feedback methods such as vibrations and touch-screen responses, enriching the overall user experience.

#### 1.2.4 Emotional loneliness relief

Emotional loneliness relief refers to the alleviation or elimination of emotional isolation and detachment experienced by individuals due to the lack of close relationships, through psychological, social, behavioral, or environmental interventions. According to Lc et al. (2023), this form of loneliness arises from unmet needs for deep social connections, understanding, and support.

To assess the impact of landscape installations on emotional loneliness relief, this study selects three evaluation metrics based on the existing literature: environmental comfort, sense of place attachment, and psychological restoration.

Ulrich (1983) points out that environmental comfort has a significant impact on an individual’s emotional state. Factors such as natural elements, the degree of greenery, and the design of landscape installations can enhance users’ sense of relaxation and pleasure, thereby helping to reduce feelings of loneliness.

Sense of place attachment also plays a key role in emotional regulation. Low and Altman (1992) argue that when individuals develop a strong sense of attachment to a particular landscape space, their emotional sense of security and comfort increases, thus alleviating feelings of loneliness.

Additionally, Kaplan and Kaplan (1989) proposed the theory of environmental restoration, suggesting that landscape design can provide individuals with opportunities for psychological restoration, helping them restore mental balance, reduce stress, and alleviate loneliness, thereby promoting positive emotional recovery.

### 1.3 Literature review and hypothesis development

According to Bongers and Mery (2011), design elements can enhance user interaction levels by increasing user engagement and interactivity. For example, interactive artworks can stimulate user interest and boost their level of participation. Bongers and Mery (2011) also point out that color, cultural elements, and innovative designs play a key role in attracting users and enhancing engagement. Additionally, research by Zhang and Um (2020) indicates that design elements not only stimulate user participation but also alleviate feelings of loneliness by strengthening psychological connections.

Although previous studies have shown that design elements influence user interaction and emotional loneliness to some extent, these studies have typically focused on other design objects, such as interactive artworks, architecture, and spatial design. They primarily emphasize the enhancement of user engagement, interactivity, and emotional connection through design elements. Landscape installations, as a specific design category, may play a distinct role in promoting social interaction and alleviating loneliness due to their unique spatial experience and environmental design. Moreover, empirical research on how different landscape installation design elements quantitatively affect user interaction and emotional loneliness relief remains limited. Therefore, this study conceptualizes the design elements of landscape installations as a second-order construct, reflected by four first-order dimensions: color, culture, function, and innovation. Based on this, the following hypotheses are proposed:

*H1*: Design elements have a significant positive impact on user interaction.

*H2*: Design elements have a significant positive impact on emotional loneliness relief.

Brough et al. (2018) argue that incorporating social interaction elements into product design, particularly designs that emphasize interactivity and social participation, can effectively alleviate emotional loneliness among the elderly population. Similarly, Wu (2023) further suggests that user interaction not only helps establish social connections among users but also significantly reduces feelings of loneliness, with this effect being particularly pronounced in the elderly. Caporaso et al. (2022) also found that interactive design helps enhance the quality of parent–child interactions, thereby strengthening emotional connections among users. Additionally, the studies by Yang et al. (2021) and Moeller (2018) support this view, both observing a positive correlation between user participation levels, interaction styles, and emotional loneliness relief.

Although existing research generally confirms a positive correlation between interactive design, user engagement, and emotional loneliness relief, these studies have mostly focused on testing direct effects and have not thoroughly explored the specific pathways through which user interaction contributes. Specifically, it remains unclear whether user interaction directly impacts the alleviation of loneliness and whether it plays a key mediating role between design elements and emotional loneliness relief. Based on this, the study proposes the following hypotheses:

*H3*: User interaction has a direct positive impact on emotional loneliness relief.

*H4*: User interaction mediates the relationship between design elements and emotional loneliness relief.

Rahmati et al. (2024) suggests in his research within social psychology that interaction style not only significantly impacts an individual’s social adaptation but also plays a prominent moderating role in social adaptation and learning contexts. This theory has the potential to extend into the field of landscape installations. Interaction style could act as a moderating variable, influencing the relationship between user interaction and emotional loneliness relief. Therefore, interaction style is not only related to an individual’s emotional response but also has the potential to play a moderating role in the interaction between users and landscape installations. Exploring its specific role as a moderating variable in landscape contexts will provide a new perspective for emotional intervention strategies in design.

Although existing research has revealed the moderating role of interaction style in social adaptation and learning contexts, and this theory holds potential for extension into other fields, the moderating role of interaction style in the “user interaction – emotional loneliness” pathway has not been empirically supported in the field of landscape installations. Therefore, this study will construct a moderation model to verify the moderating effect of interaction style in the process through which user interaction influences emotional loneliness relief. Based on this, the following hypothesis is proposed:

*H5*: Interaction style moderates the relationship between user interaction and emotional loneliness relief.

**Figure 1:**
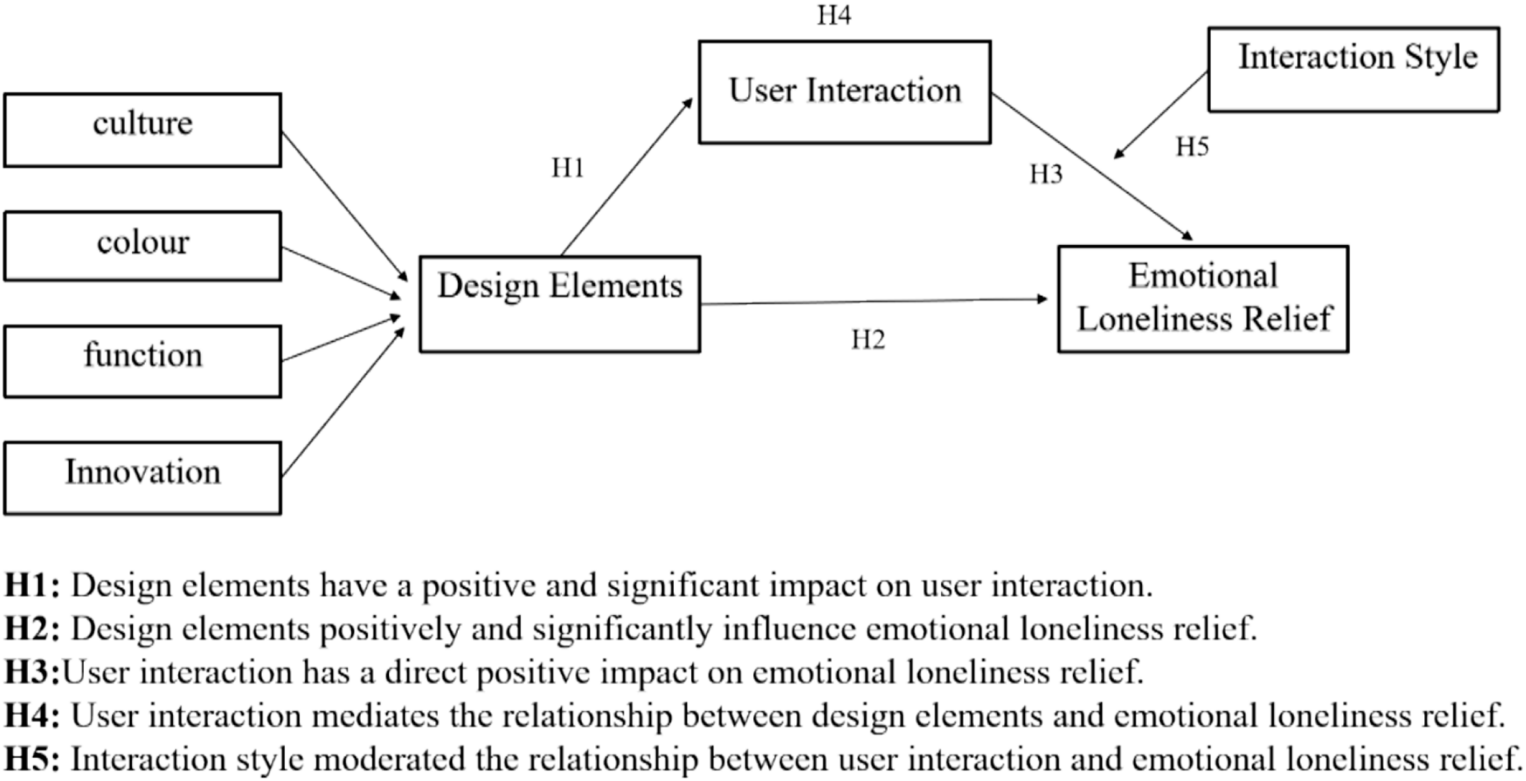
Research model.

The research model is shown in Figure 1.

## 2 Materials and methods

### 2.1 Research design

This study employs a combination of case study and questionnaire survey methods, verified through mathematical modeling. The research focuses on the landscape installations at the Han River Art Park in Seoul, with the aim of analyzing how the design elements and user interaction within the park’s landscape art installations contribute to emotional loneliness relief. The analytical methods employed in this study, including their procedures and purposes, are summarized in Table 1.

**Table 1:** Research methods analysis table.

Analysis methods	How the analysis was conducted	Findings revealed
Case Study	1. Establishing Criteria: Three clear selection criteria were set. a) Characteristics consistent with landscape installation attributes. b) Geographic accessibility. c) Clear demonstration of design elements. 2. Defining Scope: Of the 36 artworks exhibited in the park, 23 were selected as the final study sample based on the established criteria. 3. Background Research: Through literature review, the policy, cultural background, and artistic value of the research site were clarified.	1. Defined the boundaries and representativeness of the study objects, ensuring the feasibility and focus of the subsequent research. 2. Revealed the core characteristics of the study sample, providing clear and tangible research targets for the survey.
Questionnaire Survey	1. Data Collection: A survey was conducted with visitors to the selected landscape installations to gather data on design elements, interaction styles, user interaction, and emotional loneliness relief variables. 2. Data Analysis: Advanced statistical methods were employed to analyze the survey data, including: a) Mediation analysis: Examining the underlying mechanisms of the ‘design elements → user interaction → emotional loneliness relief’ pathway. b) Moderated mediation analysis: Introducing ‘interaction style’ as a moderating variable to examine whether the strength of the mediation pathway of ‘design → interaction → emotion’ differs significantly due to the interaction style of the installation.	1. Revealed the mechanisms of influence: Aimed to verify whether ‘user interaction level’ serves as a key bridge connecting design and emotional outcomes. 2. Defined the boundaries of influence: Investigated whether ‘interaction style’ acts as a ‘moderating switch’ in this mechanism, thereby revealing the universality of landscape installations’ effectiveness in alleviating loneliness for users with different interaction styles.
Mathematical Analysis	Used a mathematical model to further verify the mechanism through which design elements alleviate emotional loneliness via user interaction.	The mathematical modeling section serves as a supplementary validation of the hypotheses. It compensates for the limited sample size in the survey and addresses the insufficient support for the conclusions.

### 2.2 Research subjects and site location

According to the Reverse Navigation Search Tourist Destination Ranking (2021), the Han River Park in Seoul ranked first in domestic tourist destination preferences. From the user’s perspective, the Seoul Han River Art Park is an art and leisure space open to the public; from a policy perspective, it serves as a cultural and artistic platform that fosters new cultural value. In terms of the artistic value of the installation artworks, the Han River Art Park offers visitors a new way to experience both the Han River and art, through visual and emotional engagement, playing an important role in enhancing the area’s aesthetic appeal and artistic atmosphere. The Seoul Han River Art Park has overturned the perception that “the Han River is only a place for rest and picnics” (Hangang Art Park, 2020). Rooted in the concept of “Han River, flowing art,” the park has transformed into a space for communication and interaction. Furthermore, the numerous artworks installed in the Ichon Han River Art Park embody the imagination and participation of Seoul’s citizens, reflecting the unique cultural features of the Han River (Ichon Han River Art Park, 2020).

**Figure 2:**
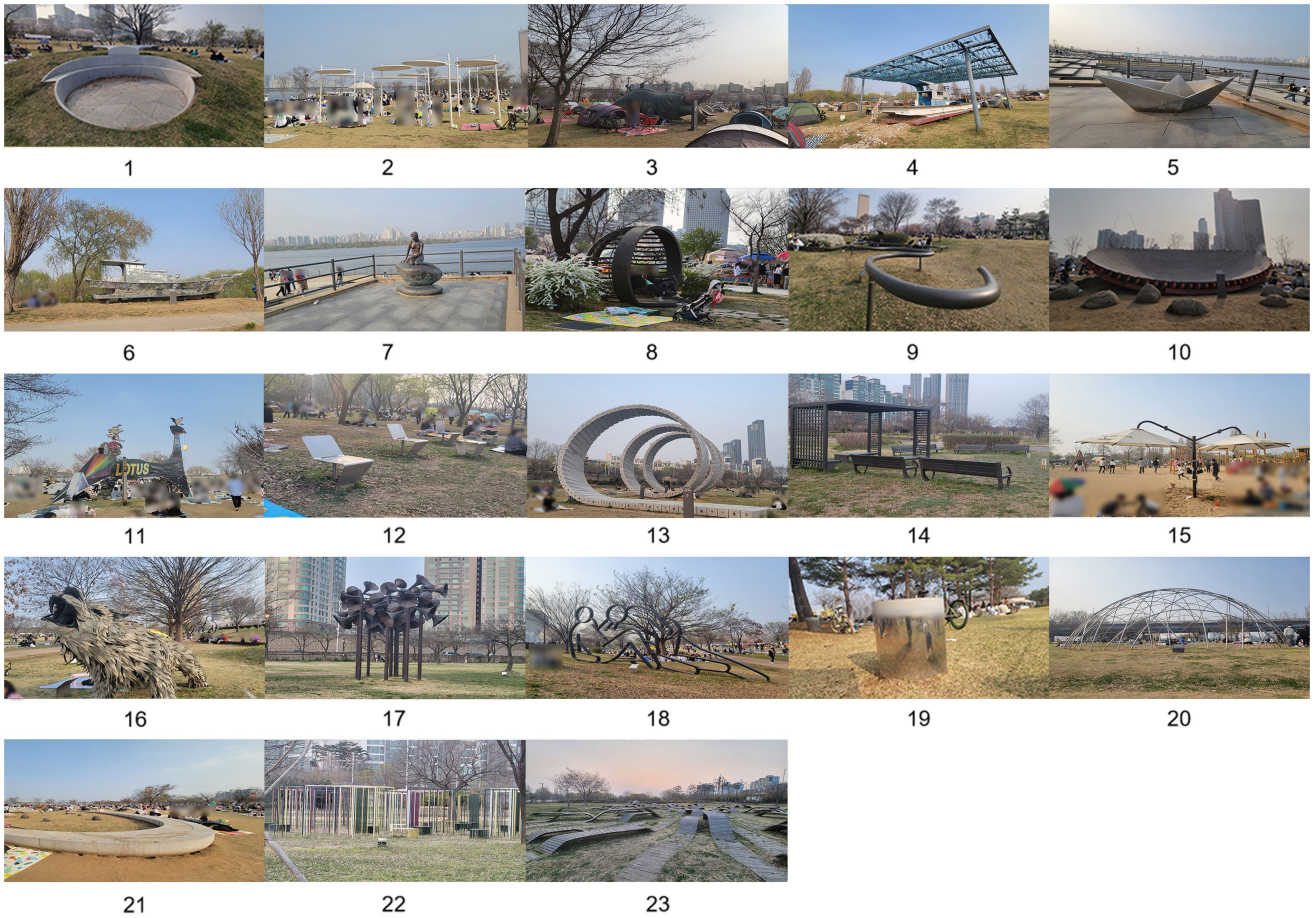
Landscape installation.

**Figure 3:**
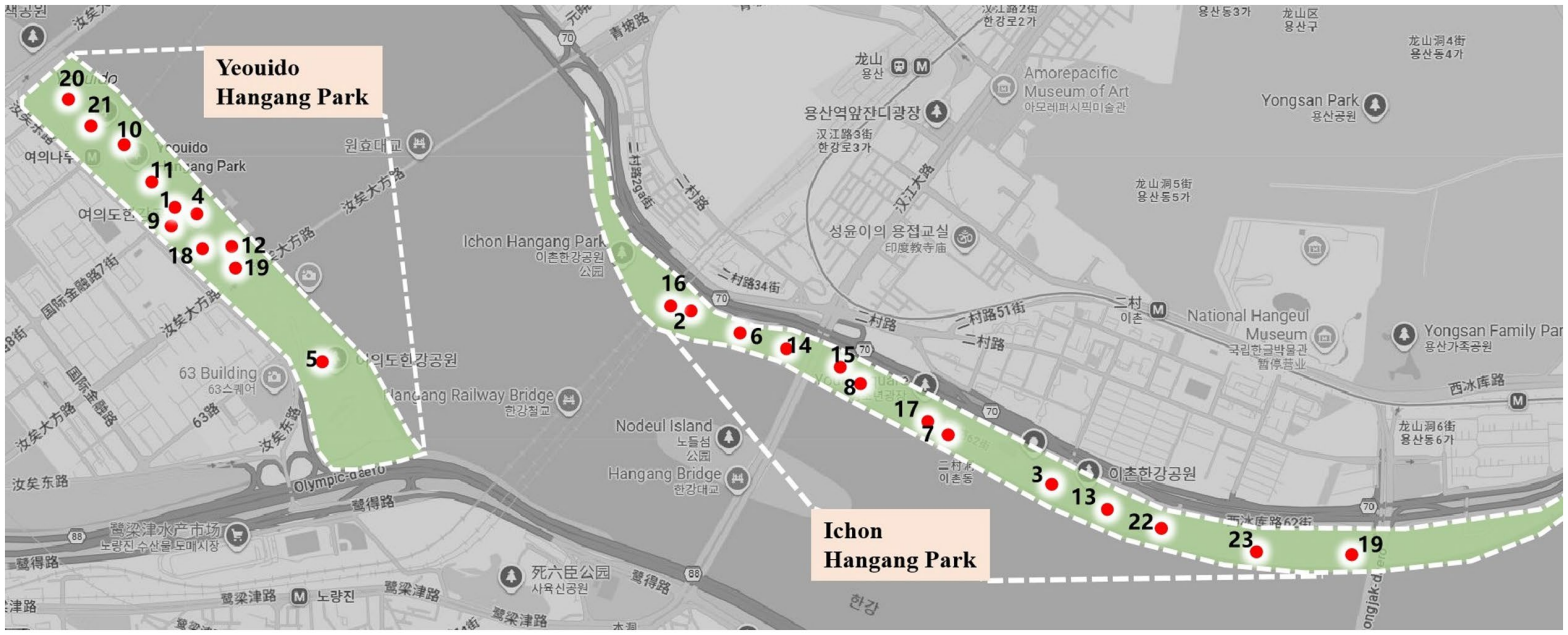
Site locations of the landscape installations.

Seoul Hangang Art Park consists of Yeouido Hangang Park located at 330 Yeouido, Yeongdeungpo, Seoul Special City, and Ichon Hangang Park located at 72nd Street 62, Ichon, Yongsan District, Seoul Special City. Since 2018, a total of 36 artworks have been exhibited at the Seoul Han River Art Park. Due to time constraints during the survey period, some artworks were not on display. To ensure the reliability of the subsequent survey, the following criteria were established for the selection of study objects: First, the artworks must align with the characteristics of landscape installations. Second, the artworks must be geographically accessible. Third, the artworks must clearly showcase the design elements. Based on these criteria, we selected the 23 landscape installation artworks from the Seoul Hangang Art Park originally documented by Gong et al., (2023), which are identified in Figure 2, and conducted in-depth research using surveys and data analysis. The geographical distribution of the research objects is shown in Figure 3.

### 2.3 Data collection and analysis methods

Joshi et al. (2015) found through their research that surveys are a widely used tool in social science research, designed to collect data from a large number of individuals through standardized questionnaires in order to analyze the relationships between variables. Therefore, this study employs a Questionnaire Survey as the primary method of data collection. Additionally, the study uses a Likert scale for scoring, with response levels ranging from “1 - Strongly Disagree” to “5 - Strongly Agree.”

The participants in the study were visitors who interacted with landscape installations at the Seoul Han River Art Park. A total of 360 questionnaires were distributed via a QR code for online completion, with 352 valid responses collected, yielding a 97.8% response rate. Among the participants, 167 were male (47.44%) and 185 were female (52.56%). The majority of respondents were aged between 19–34 years (50.57%) and 35–49 years (38.64%). Prior to distributing the questionnaires, the researchers provided a detailed explanation of the study’s objectives to the public and explicitly stated that no personal privacy information would be involved in the study, ensuring informed consent from all participants.

This study employs mediation and moderation analysis to explore the roles of user interaction and interaction style in alleviating emotional loneliness through landscape installation design elements. Through statistical analysis, we verify how design elements reduce loneliness through user interaction and reveal the enhancing role of interaction style in this process. All data analyses were conducted using SPSS statistical software.

## 3 Results

### 3.1 Reliability analysis

As shown in Table 2, except for the “Innovation” dimension, the Cronbach’s *α* coefficients for the six variables (Culture, Color, User Interaction, Emotional Loneliness Relief, Functionality, and Interaction Style) range from 0.83 to 0.88, suggesting that these variables demonstrate good measurement stability and reliability. However, the CITC value for the “Innovation” dimension is below 0.2, indicating a weak correlation with the other items. According to Peroni and Shotton (2020), when the CITC value is below 0.2, it is generally recommended to remove the item to improve overall reliability. Furthermore, the “alpha if item deleted” for the “Innovation” dimension is significantly higher than the original α value, further supporting this recommendation. According to Oi (2016), a Cronbach’s α value between 0.70 and 0.95 is considered acceptable. Therefore, the overall reliability of the scale in this study meets the required standards, making it suitable for further analysis.

**Table 2:** Reliability analysis.

Items	Corrected Item-Total Correlation (CITC)	Cronbach Alpha if Item Deleted	Cronbach *α*
CUL1	0.718	0.782	
CUL2	0.721	0.78	0.847
CUL3	0.703	0.797	
COL1	0.706	0.813	
COL2	0.726	0.794	0.854
COL3	0.743	0.778	
UI1	0.712	0.757	
UI2	0.707	0.762	0.836
UI3	0.671	0.797	
IN1	−0.102	−0.198	
IN2	−0.108	−0.178	−0.269
IN3	−0.126	−0.117	
ELR1	0.712	0.744	
ELR2	0.676	0.780	0.831
ELR3	0.682	0.774	
FUN1	0.717	0.759	
FUN2	0.692	0.784	0.838
FUN3	0.694	0.782	
IS1	0.744	0.836	
IS2	0.732	0.840	0.875
IS3	0.734	0.840	
IS4	0.718	0.846	

CUL, culture; COL, color; FUN, function; UI, user interaction; IN, innovation; ELR, emotional loneliness relief; IS, interaction style.

### 3.2 Exploratory factor analysis

Exploratory factor analysis (EFA) was conducted to test the validity of the data. As shown in Table 3, all research items have commonality values greater than 0.4, indicating that the information from these items can be effectively extracted. The KMO value is 0.878, exceeding the threshold of 0.7, which confirms the adequacy of the data for factor analysis. Furthermore, the variance explained for the six factors is as follows: 15.507, 12.249, 12.137, 12.119, 12.100, and 11.766%, with a cumulative explained variance of 75.878%, which is well above the 50% threshold, suggesting that a substantial amount of information has been extracted.

**Table 3:** Results of the validity analysis.

Items	Factor loading						Communalities
	Factor 1	Factor 2	Factor 3	Factor 4	Factor 5	Factor 6	
CUL1		0.824					0.781
CUL2		0.786					0.764
CUL3		0.827					0.769
COL1			0.770				0.744
COL2			0.839				0.796
COL2			0.831				0.797
UI1					0.853		0.786
UI2					0.831		0.765
UI3					0.791		0.721
ELR1						0.837	0.776
ELR2						0.820	0.757
ELR3						0.783	0.735
FUN1				0.816			0.776
FUN2				0.833			0.771
FUN3				0.768			0.725
IS1	0.819						0.740
IS2	0.839						0.751
IS3	0.822						0.740
IS4	0.766						0.724
Eigenvalues (Initial)	6.674	2.441	1.613	1.359	1.189	1.142	–
% of Variance (Initial)	35.126%	12.846%	8.487%	7.150%	6.257%	6.013%	–
% of *Cum*. Variance (Initial)	35.126%	47.972%	56.458	63.609%	69.865%	75.878%	–
Eigenvalues (Rotated)	2.946	2.327	2.306	2.303	2.299	2.236	–
% of Variance (Rotated)	15.507%	12.249%	12.137%	12.119%	12.100%	11.766%	–
% of *Cum*. Variance (Rotated)	15.507%	27.756%	39.892%	52.012%	64.112%	75.878%	–
KMO	0.878						–
Bartlett’s Test of Sphericity (Chi-Square)	3417.987						–
*df*	171						–
*p value*	0.000						–

CUL, culture; COL, color; FUN, function; UI, user interaction; ELR, emotional loneliness relief; IS, interaction style.

According to De Vito et al. (2019), the KMO value, which ranges from 0 to 1, assesses the suitability of the data for factor analysis, with values above 0.7 indicating a good fit. Additionally, commonality values above 0.4 suggest that the data items are appropriate for extraction, while the variance explained reflects the amount of information extracted. According to Delgado et al. (2019), factor loadings greater than 0.5 are considered valid, which aligns with the results obtained in this study.

Finally, factor loadings and item-factor correspondences were reviewed, confirming the validity of the factors. According to the analysis, Factor 1 is labeled as “Interaction Style” (IS), which includes interactions with the landscape device through touch, hearing, vision, and smell. Factor 2 is named “Culture” (CUL), incorporating traditional cultural elements, environmental protection concepts, and the ability to convey social issues. Factor 3 is “Color” (COL), referring to the strong contrast of color with the surrounding environment, moderate warmth/coolness, and high color purity. Factor 4 is “Function” (FUN), including ornamental aesthetic function, practical use, and entertainment function. Factor 5 is “User Interaction” (UI), including good sound interaction, light-shadow interaction, and dynamic interaction. Factor 6 is “Emotional Loneliness Relief” (ELR), which involves increasing opportunities for communication, fostering spiritual resonance, and providing entertainment functions for enjoyment.

### 3.3 Validated factor analysis

#### 3.3.1 Convergent validity

The convergent validity was tested by combining combined reliability (CR) and Average variance extraction (AVE). According to Hair et al. (2019), CR values are typically greater than 0.7, and AVE values are usually greater than 0.5. As shown in Table 4, all of the AVE values corresponding to each factor are greater than 0.5 and all of the CR values are higher than 0.7, implying that the data in this analysis have good convergent validity. Further analysis of the optimal number of factors for the two-order design elements, as Table 5 shows, only the model M0, i.e., the three-factor model, passes each of the test quantities, and each of the test quantities for the models M1, M2, and M3 fails to pass the test, indicating that the three-factor model is optimal.

**Table 4:** Convergent validity analysis.

Factor	Items	Factor loading	Std. Error	*z* (CR)	*p*	AVE	CR
CUL	CUL1	0.806	–	–	–		
	CUL2	0.805	0.050	19.561	0.000	0.649	0.847
	CUL3	0.804	0.051	19.526	0.000		
COL	COL1	0.811	–	–	–		
	COL2	0.809	0.050	19.770	0.000	0.661	0.854
	COL2	0.818	0.052	20.061	0.000		
FUN	FUN1	0.801	–	–	–		
	FUN2	0.790	0.050	19.033	0.000	0.633	0.838
	FUN3	0.797	0.051	19.244	0.000		
DE	CUL	0.766	–	–	–		
	COL	0.749	0.116	8.261	0.000	0.5859	0.8093
	FUN	0.781	0.124	8.329	0.000		
UI	UI1	0.804	–	–	–		
	UI2	0.811	0.067	14.602	0.000	0.630	0.836
	UI3	0.766	0.068	14.032	0.000		
ELR	ELR1	0.807	–	–	–		
	ELR2	0.762	0.068	13.907	0.000	0.621	0.831
	ELR3	0.795	0.068	14.349	0.000		
IS	IS1	0.812	–	–	–		
	IS2	0.789	0.061	15.800	0.000	0.637	0.875
	IS3	0.798	0.063	16.010	0.000		
	IS4	0.795	0.061	15.944	0.000		

AVE, average variance extracted; CR, combined reliability; CUL, culture; COL, color; FUN, function; DE, Design elements; UI, user interaction; ELR, emotional loneliness relief; IS, interaction style.

**Table 5:** Two order CFA and fit index of DE.

Serial number	Modeling	*χ*^2^/df	GFI	RMSEA	CFI	NFI	AGFI
M0	3-factor mode: CUL, COL, FUN	1.109	0.984	0.018	0.998	0.983	0.969
M1	2-factor mode: CUL, COL+FUN	10.340	0.814	0.163	0.844	0.831	0.677
M2	2-factor mode: COL, CUL + FUN	9.748	0.822	0.158	0.854	0.841	0.691
M3	1-factor mode: CUL + COL+FUN	17.101	0.733	0.214	0.721	0.710	0.555

CUL, culture; COL, color; FUN, function; DE, Design elements.

#### 3.3.2 Discriminate validity examination

The analysis of discriminant validity (Table 6) shows that for design elements, the AVE square root value is 0.765, which is greater than the absolute maximum of 0.383, implying that it has good discriminant validity; and for user interaction. The AVE square root value is 0.794, which is greater than the absolute maximum of 0.399, meaning that it has good discriminant validity. For interaction style, the AVE square root value is 0.798, which is greater than the absolute maximum of 0.429, meaning that it has good discriminant validity. For emotional loneliness relief, its AVE square root value is 0.788, which is greater than the maximum value of the absolute value of the inter-factor correlation coefficient of 0.429, implying that it has good discriminant validity.

**Table 6:** Discriminant validity: Pearson’s correlation and AVE square root value.

	DE	UI	IS	ELR
DE	**0.765**			
UI	0.340	**0.794**		
IS	0.383	0.387	**0.798**	
ELR	0.365	0.399	0.429	**0.788**

The bold diagonal elements are the square roots of each AVE; construct correlations are shown off-diagonal.

#### 3.3.3 Overall model testing

The model fit indicators are used to analyze the overall model fit validity, from Table 7, the *χ*^2^/*df* indicator value is 1.280, which is smaller than the reference value of 3, the RMSEA is 0.028, which is less than the 0.1 standard, the GFI indicator value is 0.95, which is larger than the reference value of 0.9, the NFI index indicator is 0.95, which is larger than the reference value of 0.9 (Doll et al., 1994), the CFI and TLI indicators are both larger than 0.9, and the RMSEA was 0.028, less than the standard 0.1 (Heu and Brennecke, 2023). According to the fitting criteria, all the fitting indicators meet the specification requirements, so the model can be further analyzed.

**Table 7:** Analysis of model fit indicators.

Fit induces	*χ*^2^/*df*	GFI	RMSEA	CFI	NFI	TLI
Reference point	<3	>0.9	<0.10	>0.9	>0.9	>0.9
Statistical value	1.280	0.950	0.028	0.988	0.950	0.986

*χ*^2^/df, Chi-square divided by degrees of freedom; GFI, Goodness of Fit Index; RMSEA, Root Mean Square Error of Approximation; CFI, Comparative Fit Index; NFI, Normed Fit Index; TLI, Tucker-Lewis Index.

### 3.4 Main effects analysis (path analysis)

**Figure 4:**
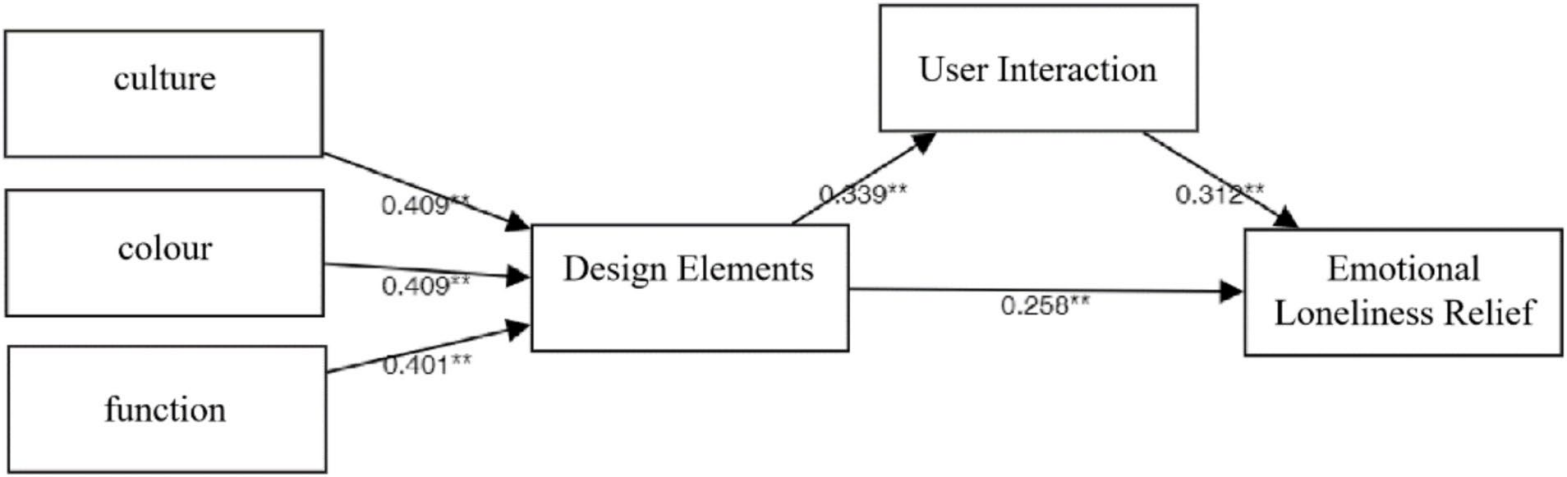
Results of path analysis.

Table 8 presents the results of the path analysis. The standardized path coefficient for the impact of design elements on user interaction is 0.339 (*z* = 6.769, *p* < 0.01), indicating a significant positive effect and supporting Hypothesis H1. The effect of design elements on emotional loneliness relief is also significant, with a standardized coefficient of 0.258 (*z* = 5.149, *p* < 0.01), supporting Hypothesis H2. Additionally, user interaction significantly contributes to alleviating emotional loneliness (*β* = 0.312, *z* = 6.229, *p* < 0.01), supporting Hypothesis H3. Furthermore, functional attributes (*β* = 0.401, *z* = 50.325, *p* < 0.01), color (*β* = 0.409, *z* = 51.701, *p* < 0.01), and cultural elements (*β* = 0.409, *z* = 51.507, *p* < 0.01) all exert significant positive effects on design components. These relationships are visually summarized in Figure 4.

**Table 8:** Hypothesized relation.

Hypothesized relation				Unstd.	*SE*	*z* (CR)	sig.	Std.	Supported?
H1	DE	→	UI	0.412	0.061	6.769	***	0.339	Supported
H2	DE	→	ELR	0.307	0.060	5.149	***	0.258	Supported
H3	UI	→	ELR	0.307	0.049	6.229	***	0.312	Supported
	FUN	→	DE	0.331	0.007	50.325	***	0.401	
	COL	→	DE	0.331	0.006	51.701	***	0.409	
	CUL	→	DE	0.331	0.006	51.507	***	0.409	

***Significant at the 1% level.

The results of this study show that design elements not only significantly promote user interaction but also directly help reduce emotional loneliness. This highlights the dual role of design in influencing both behavior and emotional experience. In addition, user interaction plays an important mediating role in relieving emotional loneliness, showing that interaction is key to improving emotional experiences. Functionality, color, and cultural elements all have significant positive effects on design elements, suggesting that a high-quality user experience relies on the combined effect of multiple design factors.

### 3.5 Mediating effects of user interaction

The analysis of mediating effects is an important aspect of statistical and psychological research that seeks to assess the role of one or more variables (mediating variables) between the independent and dependent variables. In this study, in order to assess whether user interaction mediates the relationship between design elements and emotional loneliness relief, the samples were bootstrapped using percentage bootstrapping and bias-corrected percentage bootstrapping with a confidence interval of 95%, as suggested by Tibbe and Montoya (2022). As can be seen in Table 9, The results of the data analysis showed that the explanatory strength of Model 3 for emotional loneliness relief was 21.9% (*F* = 48.877, *p* < 0.001), which was significantly improved compared with Model 1, indicating that the design elements, user interaction, had a better explanatory effect on emotional loneliness relief. After introducing user interaction into the model, the positive effect of design elements on emotional loneliness relief was reduced but still significant (*β* before = 0.435, *β* after = 0.309, *p* < 0.001), and the positive effect of user interaction was significant (*β* = 0.306, *p* < 0.001, *t* = 6.184), suggesting that user interaction plays a part in the positive effect of design elements on emotional loneliness alleviation.

**Table 9:** Mediated effects model test.

	ELR(1)	UI(2)	ELR(3)
Constant	1.821** (8.594)	1.942** (8.921)	1.227** (5.498)
DE	0.435** (7.334)	0.413** (6.775)	0.309** (5.148)
UI			0.306** (6.184)
Sample size	352	352	352
*R* ^2^	0.133	0.116	0.219
Adjustment *R*^2^	0.131	0.113	0.214
*F-*value	*F* (1,350) = 53.784, *p* = 0.000	*F* (1,350) = 45.897, *p* = 0.000	*F* (2,349) = 48.877, *p* = 0.000

*p < 0.05 **p < 0.01. *t* values are shown in parentheses.

As shown in Table 10, the mediation effect study was conducted using the percentile Bootstrap sampling test, and the results show that for the mediation path ‘design elements → user interaction → emotional loneliness relief’, the 95% interval does not include the number 0 (95% CI: 0.065 ~ 0.148), thus indicating that this mediation effect path exists, which suggests the existence of this mediating effect path, supporting H4.

**Table 10:** Mediating role test.

Path relation	Significance	Effect	95% CI		*z*/*t*	*p*	Conclusion
			Lower	Upper			
DE → UI → ELR	Indirect effect	0.126	0.065	0.148	5.93	0.000	intermediary
DE → ELR	Direct effect	0.309	0.191	0.426	5.148	0.000	
DE → ELR	Aggregate effect	0.435	0.319	0.551	7.334	0.000	

DE, Design elements; UI, user interaction; ELR, emotional loneliness relief.

### 3.6 Moderated mediation effects of emotional loneliness relief

In recent years, the Bootstrap method has been increasingly valued in mediated effects analysis due to its flexibility and weak assumption conditions on sample distribution, Therefore, in order to investigate the relationship between the influence of interaction style on user interaction and emotional loneliness relief, this study uses PROCESS model 14 to conduct a moderated mediation effect analysis. As shown in Table 11, the moderated mediation effect analysis involves 2 models, as specified in (Equations 1, 2):


(1) $$\begin{array}{l} \mathrm{ELR}=2.538+0.162\times \mathrm{DE}-0.197\times \text{IS}\\ -0.190\times \mathrm{UI}+0.130\times \mathrm{UI}\times \text{IS} \end{array}$$

(2) UI=1.942+0.413×DE

**Table 11:** Regression model summary table.

	ELR	UI
Constant	2.538** (4.357)	1.942** (8.921)
DE	0.162* (2.568)	0.413** (6.775)
IS	−0.197 (−1.244)	
UI	−0.190 (−1.249)	
UI*IS	0.130** (2.951)	
Sample size	352	352
*R* ^2^	0.288	0.116
Adjustment *R*^2^	0.278	0.111
*F-*value	*F* (4,347) = 35.175, *p* = 0.000	*F* (1,350) = 45.897, *p* = 0.000

*p < 0.05 **p < 0.01. *t* values are shown in parentheses.

**Figure 5:**
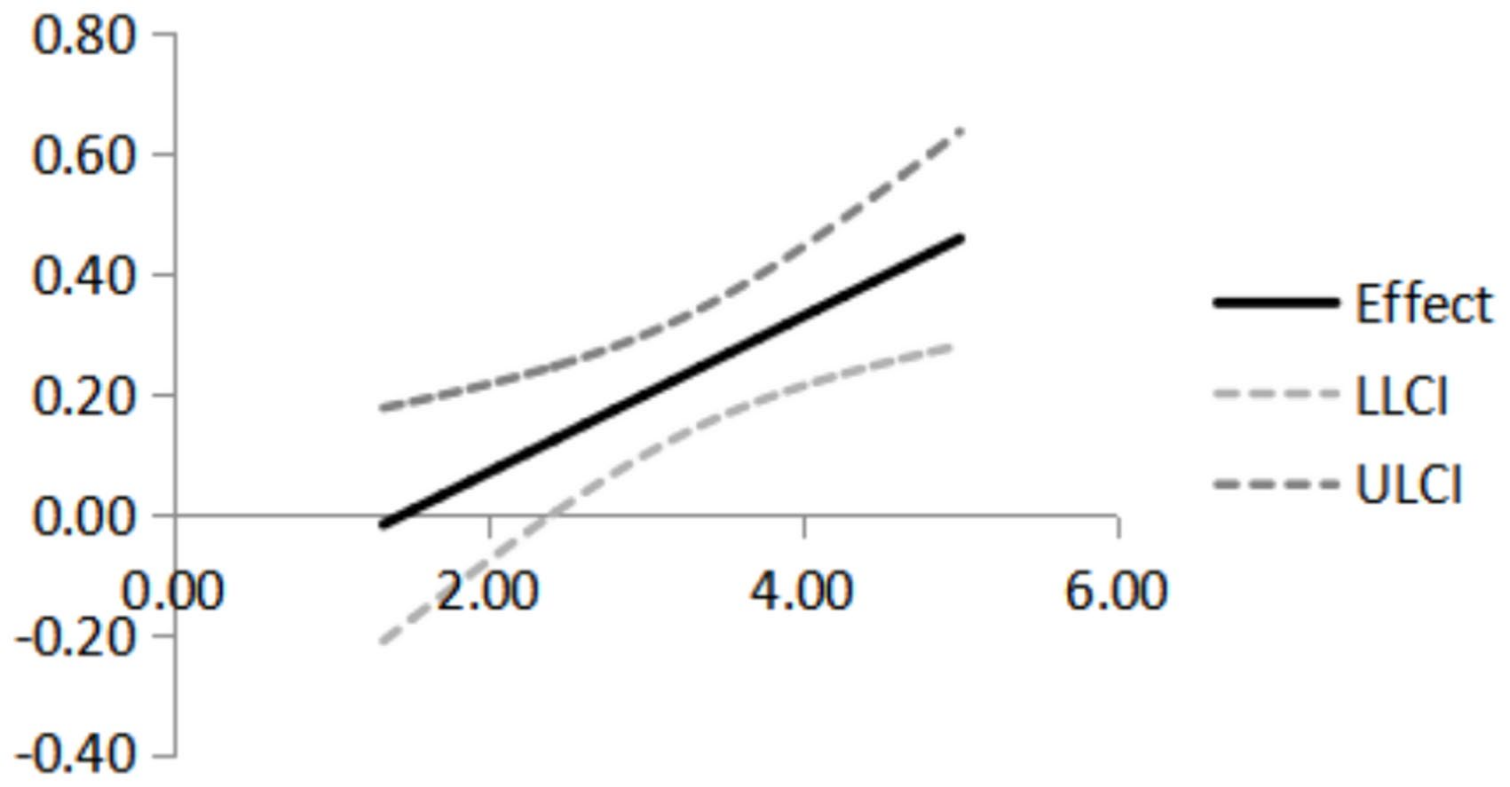
Effect of interaction style on the relation between user interaction and emotional loneliness relief.

As shown in Table 12, for the mediator variable of user interaction, at the mean and high level, the boot 95% CI does not incorporate the number 0, which means that there is mediation at this level, and the Effect value is 0.100 and 0.161. In summary, the interaction style moderate the mediating role played by user interaction between design elements and emotional loneliness relief, supporting H5. When the interaction score is low, the mediating role of user interaction is not significant, but as the interaction score is higher, the role of user interaction will be stronger. As shown in Figure 5.

**Table 12:** Conditional indirect effect results.

Intermediary variable	Level	Level value	Effect	BootSE	BootLLCI	BootULCI
UI	Low level (−1SD)	2.198	0.039	0.028	−0.016	0.093
	Average value	3.338	0.100	0.024	0.055	0.150
	High level (+1SD)	4.478	0.161	0.037	0.094	0.238

BootLLCI and BootULCI indicate the lower and upper bounds of the 95% confidence interval from percentile bootstrap sampling.

### 3.7 Mathematical analysis

Next, we conducted a precise analysis through mathematical modeling to further quantify the moderating effect of interaction style. We deeply analyzed how landscape installation design elements alleviate emotional loneliness through user interaction and interaction style, and verified the mechanism by which design elements alleviate emotional loneliness through user interaction; provide theoretical support for “experiential design”; This provides valuable theoretical basis for the psychological therapeutic benefits of landscape design and empirical evidence for the psychological health design of interaction styles in public spaces.

#### 3.7.1 Main effect model


(3) ELR=0.258.DE−0.312.UI+ϵ

#### 3.7.2 Moderating effect model

(4) ELR=0.162.DE−0.197.IS−0.190.UI+0.130.(UI.IS)+ϵ

Using the two mathematical models (Equations 3, 4), we validated the mechanism by which design elements alleviate emotional loneliness through user interaction and revealed the enhancing effect of interaction styles, providing empirical evidence for the design of mental health-supportive public spaces.

## 4 Discussion

Based on the results of path analysis, mediation effects, and moderation effects, all five hypotheses proposed in this study were statistically supported. First, the path analysis shows that design elements have a significant positive effect on user interaction (*β* = 0.339, *p* < 0.01) and also facilitate emotional loneliness relief (*β* = 0.258, *p* < 0.01), thereby supporting Hypotheses H1 and H2. Moreover, user interaction significantly directly affects emotional loneliness relief (*β* = 0.312, *p* < 0.01), thereby supporting Hypothesis H3. The study reveals a strong relationship between design elements and user interaction, which aligns with existing research in product and spatial design, such as Bongers and Mery’s interactive art pieces and the exhibition environments studied by CaPoraso et al. This suggests that landscape installations’ ability to foster user interaction is closely linked to their cultural, color, and functional design elements. Furthermore, the findings confirm that design elements are crucial in alleviating emotional loneliness (H2). When individuals perceive higher levels of design in landscape installations, they show relief from feelings of loneliness, aligning with Zhang’s findings. The results also reveal that user interaction further alleviates emotional loneliness (H3), in line with Wu and Brough’s recommendations in product design.

According to the mediation analysis, the Bootstrap test shows that the 95% confidence interval for the path ‘design elements → user interaction → emotional loneliness relief’ does not contain 0 (95% CI: 0.065–0.148), confirming that user interaction partially mediates the relationship between design elements and emotional loneliness relief, thereby supporting Hypothesis H4. This supports the previous research by Wu (2023), which indicates that user interaction not only helps to establish social connections between users but also significantly alleviates feelings of loneliness.

Based on the analysis of the moderated mediation effect, the interaction style moderates the relationship between user interaction and emotional loneliness relief. When the interaction style is at moderate or high levels, the mediating effect of user interaction is significant (Effect = 0.100–0.161), while at a low level, it is not significant, thereby supporting Hypothesis H5, in line with the views of Hassanzadeh et al. This study contributes to the existing literature by theoretical research. Firstly, our study provides evidence that design elements influence user interaction and emotional loneliness relief of landscape installations. Second, this study provides the first quantitative study of how interaction style influence the relationship between user interaction and emotional loneliness relief. Thus, this study contributes to research that reveals how interaction style influence people’s emotional states when using products.

## 5 Conclusion

The objectives of this study are to investigate the effects of design elements in landscape installations on user interaction and emotional loneliness relief. The primary contribution of this research lies in its first quantitative analysis of how design elements, such as cultural aspects, color, and function, influence the alleviation of emotional loneliness. Through survey data and statistical analysis, we confirmed the positive effects of design elements on user interaction and emotional loneliness relief, and examined the moderating role of interaction style in this process.

The empirical results of this study strongly support the proposed theoretical hypotheses. Specifically, the direct effects of design elements on user interaction and emotional loneliness relief (H1, H2) are supported. User interaction not only directly alleviates emotional loneliness (H3), but also plays a significant mediating role between design elements and emotional loneliness relief (H4). Additionally, the moderating effect of interaction style (H5) is confirmed, furthering our understanding of the process through which design mitigates emotional loneliness. Furthermore, we find that the three dimensions of function (*β* = 0.401), color (*β* = 0.409), and culture (*β* = 0.409) each make a similarly significant contribution to design elements. This result highlights the importance of not neglecting any one aspect in landscape installation design. Instead, a harmonious integration of function, aesthetics, and culture should be pursued to collectively enhance user experience. As a higher-order construct composed of function, color, and culture, design elements in landscape installations not only directly alleviate users’ emotional loneliness, but also indirectly alleviate loneliness by stimulating user interaction. This suggests that design influences user experience through both direct emotional empowerment and indirect behavioral guidance. Additionally, user interaction is confirmed as the crucial link connecting design elements and emotional experience. Its partial mediating role between design elements and emotional loneliness relief clearly elucidates the pathway through which design impacts emotions via behavior. In other words, effective landscape installations provide emotional support by fostering user engagement. Finally, this study finds that interaction style plays a crucial moderating role in this mechanism. When the interaction style matches users’ cognitive habits and preferences, the effect of user interaction on alleviating loneliness is more pronounced. Conversely, this effect weakens when the interaction style does not match. This underscores the equal importance of “how to interact” and “whether to interact” in the design of interventions for loneliness.

This study contributes theoretically by incorporating the moderating variable of interaction style into the research framework examining the impact of design on emotions, thus deepening our understanding of the “design, behavior, and emotion.” Although the findings of this study primarily apply to a specific context, the insights gained regarding design elements and user behavior can offer valuable guidance for future design research, particularly in exploring how design elements influence emotional loneliness alleviation. For instance, designers should focus on integrating function, color, and cultural elements in landscape installation design, and carefully craft intuitive and emotionally resonant interaction styles that align with these elements.

This study has certain limitations and also provides directions for future research. Firstly, the case selection is focused on the Seoul Han River Art Park. While the results provide insights into the design impact mechanisms in this specific context, the generalizability of the conclusions remains to be validated due to the geographical and cultural homogeneity. Future research could incorporate a more diverse range of geographical and cultural samples to further test the external validity of the model from a global comparative perspective. Additionally, this study employs cross-sectional data, which, while revealing immediate relationships between variables, does not capture the dynamic changes in users’ emotions and interaction behaviors. Therefore, future studies should adopt longitudinal tracking designs to more accurately reveal the sustained impact pathways and effects of landscape installations on emotional loneliness.

## Data Availability

The raw data supporting the conclusions of this article will be made available by the authors, without undue reservation.
